# Atypical Presentation of Aseptic Meningitis Due to Varicella Zoster: A Case Report

**DOI:** 10.5811/cpcem.2021.7.53596

**Published:** 2021-08-31

**Authors:** Sharon Jia, Thuyvi Luong

**Affiliations:** WellSpan York Hospital, Department of Emergency Medicine, York, Pennsylvania

**Keywords:** case report, varicella, meningitis, COVID, headache

## Abstract

**Introduction:**

Varicella zoster virus (VZV) meningitis is primarily an infection of the immuno-compromised. However, it can also affect immunocompetent individuals. Reactivation of VZV typically presents with a distinct dermatomal rash suggestive of varicella zoster, but there have also been reports of VZV meningitis presenting without a rash.

**Case Report:**

We describe a case of VZV meningitis in a healthy, 30-year-old male presenting to the emergency department shortly after receiving his first coronavirus disease 2019 vaccination. He was treated with intravenous acyclovir and then discharged home on oral valacyclovir.

**Conclusion:**

Emergency physicians should consider aseptic meningitis in immunocompetent patients presenting with atypical headaches in this population.

## INTRODUCTION

Varicella zoster virus (VZV) is part of the herpesvirus family. Initial infection causes varicella, also known as chickenpox. The virus becomes latent within the cranial nerve, dorsal root, and autonomic ganglia. Reactivation of the virus can occur as a result of decreased T cell-mediated immunity, typically causing a dermatomal vesicular rash.^1^ Major risk factors include age greater than 50, hematopoietic stem cell or organ transplant, autoimmune disease, and human immunodeficiency virus (HIV) infection. Complications of VZV reactivation include postherpetic neuralgia, herpes zoster ophthalmicus, VZV vasculopathy, and aseptic meningitis.^2^ Here we present an atypical case of VZV meningitis in an immunocompetent, 30-year-old male presenting to the emergency department following a recent coronavirus disease 2019 (COVID-19) vaccination.

## CASE REPORT

A 30-year-old, previously healthy male presented to the emergency department with a three-day history of a persistent, throbbing headache. He described the headache as worse in the morning upon awakening and exacerbated by bending over, lying down, and sneezing. The patient reported headaches in the past, but this episode had not responded to ibuprofen as usual. He denied any medical history aside from a diagnosis of Lyme disease approximately 10 years prior. This was treated with a full course of doxycycline and yielded no further complications. He had received his first COVID-19 vaccination 12 days prior to presentation, but otherwise had only taken daily ibuprofen since the onset of his headache. He denied sick contacts, recent travel, and tobacco or drug use history. Review of systems was negative for fever, changes in vision, photophobia, chest pain, shortness of breath, nausea, vomiting, diarrhea, rashes, or recent traumas.

His vital signs were within normal limits. On physical exam, pupils were equal, round, and reactive to light. The patient had no focal neurologic deficits. Cranial nerves II–XII, sensation, motor strength, speech, and coordination were intact. His neck was supple with full range of motion and no rigidity. There were no rashes or skin lesions.

Complete blood count and basic metabolic panel were unremarkable. A non-contrast computed tomography of the head revealed no evidence of acute intracranial hemorrhage or midline shift, mass, or intra-axial or extra-axial fluid collection. A lumbar puncture was performed and was significant for opening pressure of 29 centimeters (cm) water (reference range: 6–25 cm water). Cerebrospinal fluid was significant for 706 white blood cells/microliter (WBC/uL) (reference range 0–5 WBC/uL), with 95% lymphocytes; 4 red blood cells (RBC)/uL (0 RBC/uL); protein of 144 milligrams/deciliter (mg/dL) (15–45 mg/dL), and glucose 52 mg/dL (40–70 mg/dL). The polymerase chain reaction (PCR) meningitis/encephalitis panel detected VZV. The patient was empirically started on intravenous (IV) vancomycin, ceftriaxone, dexamethasone, and acyclovir. Further questioning revealed that the patient had chickenpox as a child.

The patient was hospitalized and continued treatment with high-dose IV acyclovir (10 mg/kilogram every eight hours). Blood cultures and testing for Lyme disease and HIV returned negative. After a 48-hour hospitalization, the patient requested discharge, and the infectious disease service recommended switching the patient from IV acyclovir to a 10-day course of valacyclovir (1000 mg every eight hours). The patient’s headache had resolved prior to discharge, and he was recommended to follow-up with his primary care provider (PCP). Unfortunately, we reviewed the chart a month afterward, and the patient had not followed up with his PCP.

## DISCUSSION

Varicella zoster virus reactivation causes a variety of neurologic complications including acute retinal necrosis, herpes zoster ophthalmicus, and aseptic meningitis. Advanced age and immunocompromised states are considered major risk factors for the development of herpes zoster and other manifestations of VZV reactivation. The most common presentations are a dermatomal rash and neuritis. Complications of VZV include postherpetic neuralgia and aseptic meningitis.^3^ The latter typically presents with symptoms such as headache, photophobia, nausea, and vomiting. Varicella zoster virus meningitis is rare in the young, healthy population, and other cases have been reported in the absence of the typical herpes zoster rash.^4^

Although rare, vaccines have also been associated with reactivation of VZV. This has been reported in patients who received vaccinations for hepatitis A, influenza, rabies, and Japanese encephalitis.^5^ Varicella zoster virus reactivation has also been seen in association with the COVID-19 vaccine, described in two cases of elderly patients diagnosed with herpes zoster within a week after receiving their vaccinations.^6^,^7^

Varicella zoster virus can be diagnosed clinically with the presence of a dermatomal rash. It can also be diagnosed in the laboratory by using PCR to analyze vesicular skin material and cerebrospinal fluid.^8^ According to Infectious Diseases Society of America guidelines, IV acyclovir is the drug of choice to treat VZV meningoencephalitis.^9^ Ganciclovir can be an alternative agent.^9^ However, IV acyclovir is not always well tolerated and has been associated with increased risk of neurotoxicity. Whereas oral acyclovir is limited by its bioavailability, oral valacyclovir is converted to acyclovir in vivo and has a three- to five-fold increase in acyclovir bioavailability.^10^ Oral valacyclovir provides therapeutic cerebrospinal-fluid acyclovir levels and inhibits most VZV strains.^11^,^12^

**CPC-EM Capsule**

What do we already know about this clinical entity?

*Reactivation of varicella zoster virus (VZV) can cause meningitis, and typically affects older and immunocompromised patients and presents with a rash.*

What makes this presentation of disease reportable?

*Our case describes an uncommon presentation of aseptic meningitis secondary to VZV in a healthy, young adult without a rash.*

What is the major learning point?

*It is important to consider aseptic meningitis on the differential when evaluating a patient who presents with an atypical headache.*

How might this improve emergency medicine practice?

*Because VZV meningitis is rarely seen in the immunocompetent population, this case report adds additional information for a better understanding of VZV.*

Case reports have described successfully treating patients for VZV meningitis with IV acyclovir and transitioning to oral valacyclovir.^13^ However, oral valacyclovir by itself does not appear to be a suitable agent as herpes zoster can progress to meningitis despite oral valacyclovir.^15^ After an appropriate dose of IV acyclovir, valacyclovir can be a suitable option for treating minimally symptomatic patients in the outpatient setting, such as the young man in this case report.

## CONCLUSION

While rare, aseptic meningitis secondary to VZV can occur in young, healthy, immunocompetent patients and can present without a rash. Emergency physicians should consider this diagnosis for atypical headaches in this population. Once the diagnosis is made, it would be prudent to initiate IV acyclovir and have the inpatient team determine whether to transition to oral valacyclovir.

## References

[1] Mueller NH, Gilden DH, Cohrs RJ (2008). Varicella zoster virus infection: clinical features, molecular pathogenesis of disease, and latency. Neurol Clin.

[2] Yawn BP, Saddier P, Wollan PC (2007). A population-based study of the incidence and complication rates of herpes zoster before zoster vaccine. Mayo Clin Proc.

[3] Nagel MA, Gilden D (2013). Complications of varicella zoster virus reactivation. Curr Treat Options Neurol.

[4] Fadhel M, Campbell N, Patel S (2019). Varicella zoster meningitis with hypoglycorrhachia on cerebrospinal fluid (CSF) analysis in a young immunocompetent host without a rash. Am J Case Rep.

[5] Walter R, Hartmann K, Fleisch F (1999). Reactivation of herpesvirus infections after vaccinations?. Lancet.

[6] Eid E, Abdullah L, Kurban M (2021). Herpes zoster emergence following mRNA COVID-19 vaccine. J Med Virol.

[7] Bostan E, Yalici-Armagan B (2021). Herpes zoster following inactivated COVID-19 vaccine: a coexistence or coincidence?. J Cosmet Dermatol.

[8] Gershon AA, Breuer J, Cohen JI (2015). Varicella zoster virus infection. Nat Rev Dis Primers.

[9] Tunkel AR, Glaser CA, Bloch KC (2008). The management of encephalitis: clinical practice guidelines by the Infectious Diseases Society of America. Clin Infect Dis.

[10] Beutner KR, Friedman DJ, Forszpaniak C (1995). Valaciclovir compared with acyclovir for improved therapy for herpes zoster in immunocompetent adults. Antimicrob Agents Chemother.

[11] Cunha BA, Baron J (2017). The pharmacokinetic basis of oral valacyclovir treatment of herpes simplex virus (HSV) or varicella zoster virus (VZV) meningitis, meningoencephalitis or encephalitis in adults. J Chemother.

[12] Lycke J, Malmeström C, Ståhle L (2003). Acyclovir levels in serum and cerebrospinal fluid after oral administration of valacyclovir. Antimicrob Agents Chemother.

[13] Gnoni M, Zaheer K, Vasser MM (2018). Varicella zoster aseptic meningitis: report of an atypical case in an immunocompetent patient treated with oral valacyclovir. IDCases.

[14] Bateman R, Naples R (2021). Herpes zoster meningitis in a young, immunocompetent adult. J Emerg Med.

